# Estimating the burden of foodborne diseases in Japan

**DOI:** 10.2471/BLT.14.148056

**Published:** 2015-06-01

**Authors:** Yuko Kumagai, Stuart Gilmour, Erika Ota, Yoshika Momose, Toshiro Onishi, Ver Luanni Feliciano Bilano, Fumiko Kasuga, Tsutomu Sekizaki, Kenji Shibuya

**Affiliations:** a Department of Veterinary Medical Science, University of Tokyo, Tokyo, Japan.; b Department of Global Health Policy, Graduate School of Medicine, University of Tokyo, 7-3-1, Hongo, Bunkyo-ku, Tokyo 113-0033, Japan.; c Department of Health Policy, National Centre for Child Health and Development, Tokyo, Japan.; d National Institute of Health Sciences, Tokyo, Japan.; e Faculty of Economics, Kyushu University, Fukuoka, Japan.; f Research Centre for Food Safety, University of Tokyo, Tokyo, Japan.

## Abstract

**Objective:**

To assess the burden posed by foodborne diseases in Japan using methods developed by the World Health Organization’s Foodborne Disease Burden Epidemiology Reference Group (FERG).

**Methods:**

Expert consultation and statistics on food poisoning during 2011 were used to identify three common causes of foodborne disease in Japan: *Campylobacter* and *Salmonella* species and enterohaemorrhagic *Escherichia coli* (EHEC). We conducted systematic reviews of English and Japanese literature on the complications caused by these pathogens, by searching Embase, the Japan medical society abstract database and Medline. We estimated the annual incidence of acute gastroenteritis from reported surveillance data, based on estimated probabilities that an affected person would visit a physician and have gastroenteritis confirmed. We then calculated disability-adjusted life-years (DALYs) lost in 2011, using the incidence estimates along with disability weights derived from published studies.

**Findings:**

In 2011, foodborne disease caused by *Campylobacter* species, *Salmonella* species and EHEC led to an estimated loss of 6099, 3145 and 463 DALYs in Japan, respectively. These estimated burdens are based on the pyramid reconstruction method; are largely due to morbidity rather than mortality; and are much higher than those indicated by routine surveillance data.

**Conclusion:**

Routine surveillance data may indicate foodborne disease burdens that are much lower than the true values. Most of the burden posed by foodborne disease in Japan comes from secondary complications. The tools developed by FERG appear useful in estimating disease burdens and setting priorities in the field of food safety.

## Résumé

**Objectif:**

Évaluer la charge des maladies d'origine alimentaire au Japon, à l'aide de méthodes développées par le Groupe de travail de référence de l'OMS sur l’épidémiologie des maladies d’origine alimentaire (FERG).

**Méthodes:**

Des avis d'experts et des statistiques sur les intoxications alimentaires pour l'année 2011 ont été utilisés pour identifier les trois principales causes des maladies d'origine alimentaire au Japon : à savoir les espèces *Campylobacter*, *Salmonella* et *Escherichia coli *entérohémorragique (ECEH). Nous avons procédé à des revues systématiques de la littérature anglaise et japonaise sur les complications causées par ces agents pathogènes, en faisant des recherches dans Embase (base de données bibliographiques de la société médicale du Japon) et Medline. Nous avons évalué l'incidence annuelle de la gastro-entérite aiguë à partir des données de surveillance disponibles, sur la base des probabilités estimées qu'une personne affectée ira consulter un médecin et sera diagnostiquée comme souffrant de gastro-entérite. Nous avons ensuite calculé les AVCI (années de vie corrigées du facteur incapacité) perdues en 2011, en utilisant les évaluations d'incidence ainsi que les coefficients de pondération de l'incapacité tirés des études publiées.

**Résultats:**

En 2011, au Japon, les maladies d'origine alimentaire causées par les espèces *Campylobacter*, *Salmonella* et ECEH ont respectivement entraîné une perte estimée à 6 099, 3 145 et 463 AVCI. Ces charges estimées sont fondées sur la méthode de reconstruction de la pyramide de surveillance. Elles sont largement liées à la morbidité -plutôt qu'à la mortalité- et sont très supérieures à celles indiquées par les données de surveillance de routine.

**Conclusion:**

Il est possible que les données de surveillance de routine reflètent des chiffres largement inférieurs à la réalité. La charge des maladies d'origine alimentaire au Japon est principalement liée à leurs complications secondaires. Les outils développés par le FERG semblent être utiles pour évaluer les charges des maladies et définir les priorités en matière de sécurité sanitaire des aliments.

## Resumen

**Objetivo:**

Evaluar la carga que plantean las enfermedades de transmisión alimentaria en Japón mediante la utilización de métodos desarrollados por el Grupo de Referencia sobre Epidemiología de la Carga de Enfermedades de Transmisión Alimentaria (FERG) de la Organización Mundial de la Salud.

**Métodos:**

Se utilizaron consultas de expertos y estadísticas en intoxicación alimentaria durante 2011 para identificar tres causas comunes en las enfermedades de transmisión alimentaria en Japón: las bacterias *Campylobacter*, *Salmonella* y *E. coli* enterohemorrágica (EHEC). Se llevaron a cabo revisiones sistemáticas de bibliografía inglesa y japonesa sobre las complicaciones causadas por estos patógenos buscando en Embase, la base de datos de la sociedad médica japonesa, y Medline. Se estimó la incidencia anual de gastroenteritis aguda de los datos de vigilancia informados, en base a probabilidades estimadas de que una persona afectada acudiría a un médico y se le confirmaría la gastroenteritis. Entonces se calcularon los años de vida ajustados en función de la discapacidad (AVAD) perdidos en 2011, utilizando los cálculos de incidencia junto con los pesos de la discapacidad derivados de estudios publicados.

**Resultados:**

En 2011, las enfermedades de transmisión alimentaria causadas por las bacterias *Campylobacter*, *Salmonella *y EHEC condujeron a una pérdida de 6.099, 3.145 y 363 AVAD, respectivamente. Estas cargas estimadas están basadas en el método de reconstrucción de la pirámide de vigilancia, se deben en gran parte a la morbilidad más que a la mortalidad y son mucho más altas que aquellas indicadas por los datos obtenidos a partir de la vigilancia rutinaria.

**Conclusión:**

Los datos de la vigilancia rutinaria pueden indicar que las cargas de enfermedades de transmisión alimentaria son mucho más bajas que los valores reales. La mayoría de la carga que plantean las enfermedades de transmisión alimentaria en Japón proviene de complicaciones secundarias. Las herramientas desarrolladas por el FERG parecen útiles a la hora de estimar las cargas de enfermedades y de configurar prioridades en el área de la seguridad alimentaria.

## ملخص

**الغرض:**

تقييم العبء الذي يفرضه المرض المنقول عن طريق الغذاء في اليابان باستخدام الطرائق التي وضعها الفريق المرجعي المعني بالوبائيات المتعلقة بعبء الأمراض المنقولة عن طريق الغذاء (FERG) التابع لمنظمة الصحة العالمية.

**الطريقة:**

تمت الاستعانة بمشورة الخبراء والإحصائيات المتعلقة بالتسمم الغذائي خلال عام 2011 لتحديد ثلاثة أسباب شائعة للإصابة بالمرض المنقول عن طريق الغذاء في اليابان: أنواع بكتريا *الكامبيلوباكتر * و *السالمونيلا * و *الإشريكية القولونية المعوية النزفية * (EHEC). وأجرينا مراجعات منهجية للكتابات الصادرة باللغتين الإنجليزية واليابانية التي تتناول المضاعفات الناتجة عن مسببات الأمراض المذكورة، وذلك عن طريق البحث في قاعدة معطيات Embase، وقاعدة معطيات ملخصات المنشورات التابعة للجمعية الطبية اليابانية، وقاعدة معطيات Medline. وأجرينا تقديرًا لنسبة حالات الإصابة بالتهاب المعدة والأمعاء الحاد سنويًا من خلال بيانات الرصد التي تم الإبلاغ عنها، وذلك بناءً على الاحتمالات التقديرية التي تفترض زيارة الشخص المصاب للطبيب والتأكد من إصابته بالتهاب المعدة والأمعاء. ومن ثم احتسبنا الخسارة التي تشير إليها سنوات العمر المصححة باحتساب مدد العجز (DALYs) في عام 2011، وذلك باستخدام تقديرات حالات الإصابة بالإضافة إلى نتائج مقياس العجز المستمدة من الدراسات المنشورة.

**النتائج:**

أدت الإصابة بالمرض المنقول عن طريق الغذاء والناتج عن وجود أنواع بكتريا *الكامبيلوباكتر *و *السالمونيلا * و *EHEC * إلى خسارة 6099، و3145، و463 عامًا بالرجوع إلى DALYs على التوالي. وتعتمد الأعباء المقدَّرة على طريقة إعادة الهيكلة الهرمية للرصد، وتعود إلى حد كبير إلى الاعتلال بدلاً من الوفيات، كما ترتفع نسبتها إلى حد بعيد عن تلك التي أشارت إليها بيانات الرصد الروتيني.

**الاستنتاج:**

قد تشير بيانات الرصد الروتيني إلى الانخفاض الشديد في معدلات الإصابة بالمرض المنقول عن طريق الغذاء مقارنةً بالقيم الحقيقية. وينشأ الجزء الأعظم من العبء الذي يفرضه المرض المنقول عن طريق الغذاء في اليابان عن مضاعفات ثانوية. وتبدو الأدوات التي وضعها FERG مفيدة لتقدير أعباء المرض ووضع أولويات في مجال سلامة الأغذية.

## 摘要

**目的:**

旨在使用世界卫生组织食源性疾病负担流行病学参考组 (FERG) 制定的方法，评估日本食源性疾病所带来的负担。

**方法:**

利用 2011 年有关食品中毒的专家会诊和统计数据，确定日本食源性疾病的三种常见致因：*弯曲杆菌和沙门氏病菌*和肠出血性*大肠杆菌 *(EHEC)。我们通过搜索 Embase、日本医学社会摘要数据库和 Medline，系统查看了有关这些病原体所导致的并发症的英文和日文文献。我们依据估计感染者前去就诊并确诊患有肠胃炎的概率，利用报告的监测数据估算出每年急性肠胃炎的发病率。然后，我们基于来自公布数据的发病率估测值以及伤残权重，计算出 2011 年损失的伤残调整寿命年 (DALY)。

**结果:**

2011 年，*因弯曲杆菌和沙门氏病菌*和 EHEC 引发的食源性疾病导致分别估计损失 6099、3145 和 463 DALY。依据金字塔重构方法，大多基于发病率而非死亡率估算出负担，其远远超出常规监测数据指示的数值。

**结论:**

常规监测数据指示的食源性疾病负担可能远远低于真实数值。日本食源性疾病带来的大多数负担源于继发性并发症。FERG 开发的工具有助于估算疾病负担及确定食品安全领域的优先事项。

## Резюме

**Цель:**

Оценка бремени болезней пищевого происхождения в Японии с применением методов, разработанных Справочной группой Всемирной организации здравоохранения по эпидемиологии бремени болезней пищевого происхождения (FERG).

**Методы:**

С помощью экспертных консультаций и статистических данных по пищевым отравлениям за 2011 год были определены три основные причины болезней пищевого происхождения в Японии: бактерии *Campylobacter* и *Salmonella*, а также энтерогеморрагический штамм кишечной палочки *Escherichia coli* (EHEC). Был проведен систематический обзор английской и японской литературы по осложнениям, вызванным данными патогенными микроорганизмами, которая была найдена в базах данных Embase, Medline и реферативной базе данных публикаций медицинского сообщества Японии. Оценивался уровень ежегодной заболеваемости острым гастроэнтеритом по данным эпиднадзора. Для его оценки использовалась расчетная вероятность того, что заболевшие придут к врачу и врач поставит диагноз «гастроэнтерит». Затем рассчитывалось количество лет жизни, утраченных в связи с болезнью (DALY) в 2011 году. Для этого использовалась оценка количества случаев заболевания и весовые коэффициенты для нетрудоспособности, полученные из опубликованных исследований.

**Результаты:**

В 2011 году болезни пищевого происхождения, вызванные бактериями *Campylobacter*, *Salmonella* и EHEC, привели к потере 6099, 3145 и 463 лет жизни, утраченных в связи с болезнью, соответственно. Выявленное бремя болезней, рассчитанное путем реконструкции пирамиды наблюдений, в большей степени связано с заболеваемостью, а не со смертностью. Оно оказалось намного выше показателей, полученных в результате обычного эпиднадзора.

**Вывод:**

Данные обычного эпиднадзора могут свидетельствовать о бремени болезней пищевого происхождения, которое намного ниже его истинных значений. Большая часть бремени болезней пищевого происхождения в Японии приходится на вторичные осложнения. Методы, разработанные FERG, оказались эффективными для оценки бремени болезней и определения приоритетов в сфере безопасности пищевых продуктов.

## Introduction

There have been few attempts to provide comprehensive, consistent and comparable estimates of the burden of acute foodborne diseases.^1^ In 2006, however, the World Health Organization (WHO) set up the Foodborne Disease Burden Epidemiology Reference Group (FERG) specifically to produce such estimates.^2^ FERG aims to provide the data and tools needed to set appropriate, evidence-informed priorities for food safety at country level. Since its launch, FERG has established several task forces that focus on parasitic and enteric diseases, chemicals and natural toxins, source attribution, computational modelling and country studies. The members of the country studies task force were asked to develop methods for estimating the burden posed by foodborne disease at national level. These methods were intended to facilitate the collection of national data on foodborne disease burdens and support the use of such data for policy-making and practice in food safety.^3^ FERG selected Albania, Japan, Thailand and Uganda as the locations for initial pilot studies estimating disability-adjusted life-years (DALYs) lost as a result of foodborne disease.^4^^,^^5^

In Japan, priorities for foodborne disease prevention are primarily based on the apparent public health significance of each disease, although impact on the food market, consumers’ risk perceptions and public opinion are also taken into consideration.^6^ The Japanese Food Sanitation Act and Infectious Disease Control Act require collection of data on the incidence of food poisoning and infectious diseases, respectively. However, as there has never been a comprehensive, internally consistent and robust assessment of the burden posed by foodborne disease in Japan, robust and objective standards for ranking priorities are lacking. Surveillance data are not as useful as formal estimates when identifying and ranking diseases in terms of their contributions to the country’s overall burden. Our objective is to assess the burden posed by common foodborne diseases in Japan, using the methods recommended by FERG and expressing the main findings in terms of DALYs.

## Methods

### Disease selection

After analysis of food poisoning statistics and consultation with experts, we identified *Campylobacter* species, *Salmonella* species and enterohaemorrhagic *Escherichia coli* (EHEC) as the first, second and third most common causes of foodborne disease in Japan in 2011.^7^ This ranking was entirely based on clinical cases in health facilities. To estimate the relative burden posed by each of these three causes of foodborne disease, we used a pyramid reconstruction method and supplemented routine surveillance and reporting data with information from telephone and patient surveys.

### Data sources

We used data from four sources to estimate the annual incidence of acute gastroenteritis caused by *Campylobacter, Salmonella* and EHEC and to estimate associated mortality rates. The four data sources were: (i) food poisoning statistics that had been compiled using information collected by local governments on outbreaks of food poisoning; (ii) surveillance data on EHEC (routine collection of data on EHEC cases in Japan was not made a legal requirement until 1999; disease caused by *Salmonella* or *Campylobacter* species was not recorded);^8^^,^^9^ (iii) national patient surveys for 1996, 1999, 2002, 2005, 2008 and 2011. (These surveys record patients in hospitals and clinics on a single day in October, coded according to the International Classification of Diseases [ICD-10]);^10^ and (iv) vital registration records assimilated by the Japan Ministry of Health, Labour and Welfare.^11^

### Incidence estimation

Because of the limitations of the reported statistics, the annual numbers of cases of acute gastroenteritis attributable to foodborne disease caused by *Campylobacter* (*Y*_1_), *Salmonella* (*Y*_2_) and EHEC (*Y*_3_) were estimated using the formulae:
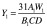
(1)

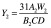
(2)

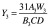
(3)where 31 represents the number of days in October. *A_i_* represents the corresponding reported incidence – *A_1_* and *A_2_* estimated from the patient survey data and disease durations^12^ and *A_3_* derived from the data collected from infectious disease surveillance. *W_i_* represents the proportions of infection attributable to foodborne disease. *B_i_* represents seasonality – calculated as the number of cases of acute gastroenteritis caused by *Campylobacter* or *Salmonella* on survey days divided by the corresponding daily mean numbers of cases of acute gastroenteritis caused by *Campylobacter* and *Salmonella* recorded in the survey years. *C* represents the proportion of incident cases confirmed by stool examination. *D* represents the proportion of incident cases who visited a physician. Data for the estimation of *C* and *D* were derived from population-based telephone surveys.^13^^,^^14^

We used a Bayesian method to estimate the probability distributions of *B_i_*, *C* and *D*. We assumed that *C* and *D* followed binomial probability distributions with a beta prior distribution for the binomial probability parameter. Because the beta prior is the conjugate distribution of the binomial likelihood, the posterior distribution is also beta-distributed.^15^ We assumed a uniform prior distribution – i.e. a special case of the beta distribution in which the probability parameter lies between 0 and 1.^14^ Once we had obtained three beta distributions, we assumed that the parameters underlying them were mutually independent and used Mathematica version 8 (Wolfram Research, Hanborough, United Kingdom of Great Britain and Northern Ireland) to calculate the distribution as the product of the three independent distributions.

Finally, the proportions (*W_i_*) of *Y_1_*, *Y_2_* and *Y_3_* attributable to foodborne disease were estimated using an expert elicitation process similar to that done in the Netherlands.^16^ We invited contributions to this estimation from experts from different scientific backgrounds – microbiology, epidemiology and food science. We invited 88 experts and thirty (34.1%) agreed to participate. We asked the experts to provide their best estimate of the percentages of individuals with gastroenteritis caused by *Campylobacter*, *Salmonella* or EHEC that had become infected by each of five pathways: food, environment, animal–human, human–human and travel. We also asked the experts to estimate the 90% confidence limits around their best estimates. Individual expert opinions were represented in terms of a Dirichlet distribution. Where more than one expert provided an opinion on the same pathway we combined the estimates using a Bayesian update method with equal weighting (details available from the corresponding author).

### Complications

In our investigation of the burden caused by complications of gastroenteritis, we used outcome trees based on a European study.^17^ The complications resulting from *Campylobacter* included Guillain-Barré syndrome, inflammatory bowel disease and reactive arthritis; from *Salmonella*, inflammatory bowel disease and reactive arthritis and from EHEC, haemorrhagic colitis and haemolytic-uraemic syndrome.^17^^,^^18^

**Fig. 1:**
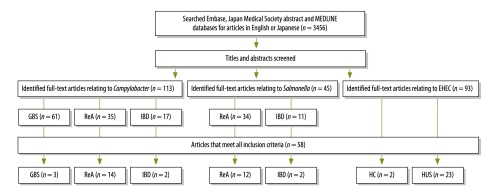
Flowchart for the selection of studies included in the systematic review on the disability associated with foodborne disease

EHEC: enterohaemorrhagic *Escherichia coli*; GBS: Guillain-Barré syndrome; HC: haemorrhagic colitis; HUS: haemolytic uraemic syndrome; IBD: inflammatory bowel disease; ReA: reactive arthritis.

We used systematic reviews of prospective cohort studies to estimate the proportions of these complications that could be attributed to gastroenteritis caused by each infectious agent. We searched the Japan medical abstract society database and Embase for relevant articles published between 1 January 1983 and 29 February 2012 and Medline for relevant articles published between 1 January 1946 and 29 February 2012.^19^ The search terms were designed by an information specialist using the appropriate medical subheadings (available from the corresponding author). We included prospective cohort studies that described, in English or Japanese, the proportions of laboratory-confirmed sequelae that resulted from gastroenteritis caused by *Campylobacter, Salmonella* or EHEC. We only used published data and made no attempt to obtain any further data from the authors of relevant articles. We excluded case reports, review papers, letters, comments, conference proceedings, studies with insufficient information on criteria, studies that only provided aggregated data for multiple conditions and unpublished studies (Fig. 1).

The title, abstract and, if appropriate, the full text of each eligible article of potential interest were screened by two authors independently. Discrepancies were resolved by discussion and consensus. We collected information on the year of publication, study duration, country and area, data source or sources, follow-up period, sample size, serotype, age group, sex, case definition and the incidence of sequelae and their associated standard errors. We assessed the quality of each included study using the Newcastle-Ottawa scale.^20^

### Data analysis

Meta-analyses of the proportions of sequelae attributable to gastroenteritis caused by *Campylobacter, Salmonella* or EHEC were done to generate pooled values of prevalence with 95% uncertainty intervals. Heterogeneity among studies was estimated using Cochran’s *Q* and the *I*^2^ statistic. Either the Freeman-Tukey double arcsine transformation or log–normal random-effects were used to stabilize model variances.^21^^–^^69^ Potential sources of heterogeneity were investigated further by analysis of subgroups by age and methods of laboratory confirmation. We used random-effects models^70^ in Stata version 13 (StataCorp. LP, College Station, United States of America).

### Estimation of mortality

Data on gastroenteritis-related deaths caused by *Campylobacter*, *Salmonella* or EHEC *–* (ICD-10 codes A045, A02 and A043 respectively) and sequelae such as Guillain-Barré syndrome, inflammatory bowel disease or haemolytic-uraemic syndrome (ICD-10 codes G610, K50/K51 and D59.3 respectively) were obtained from the Japan vital registration system.^11^ These mortality estimates were adjusted based on the proportions estimated to be attributable to foodborne disease. We did not adjust for possible misclassification.

### Estimation of burden

We used DALYs to assess the burden of foodborne disease caused by *Campylobacter*, *Salmonella* or EHEC in Japan in 2011. DALYs combine the years of potential life lost due to premature death with the years lived with disabilities.^71^ We estimated years of potential life lost by multiplying the number of deaths due to a particular form of foodborne disease by the number of potential life-years lost due to premature death from that disease. The latter was based on standard life expectancies from the Global Burden of Disease (GBD) 2010 study.^72^ The corresponding years lived with disabilities were calculated as the product of the number of incident cases of a particular form of foodborne disease, the mean duration of that disease and the disability weight for that disease. Age-specific disease incidences were estimated from the age distributions recorded in food poisoning and infectious diseases statistics for Japan. Whenever possible, we used disease durations and disability weights from studies conducted in Europe.^17^^,^^18^ To be consistent with the assumptions made in the GBD 2010 study, we did not apply any discounting or non-uniform age-weighting. DALY components were calculated separately for each sex and age group and then summed to obtain estimates of the total burdens.

### Uncertainty analysis

Uncertainty intervals were derived by Monte-Carlo simulation within the R statistical package (R Foundation for Statistical Computing, Vienna, Austria). Appropriate probability distributions were specified for parameters that, based on the published literature, were considered to be important sources of uncertainty. Estimates were repeatedly calculated from randomly drawn sets of input values, and 95% uncertainty intervals were derived from the 2.5th and 97.5th percentiles of the output values. The process was continued until the difference between the means of the incremental iterations satisfied the stopping criterion of less than 1 unit difference in the mean of the outcome estimates. The number of draws ranged from 22, for acute gastroenteritis caused by *Campylobacter*, to 52 951, for inflammatory bowel disease caused by *Salmonella*.

## Results

### Incidences of acute gastroenteritis

**Fig. 2:**
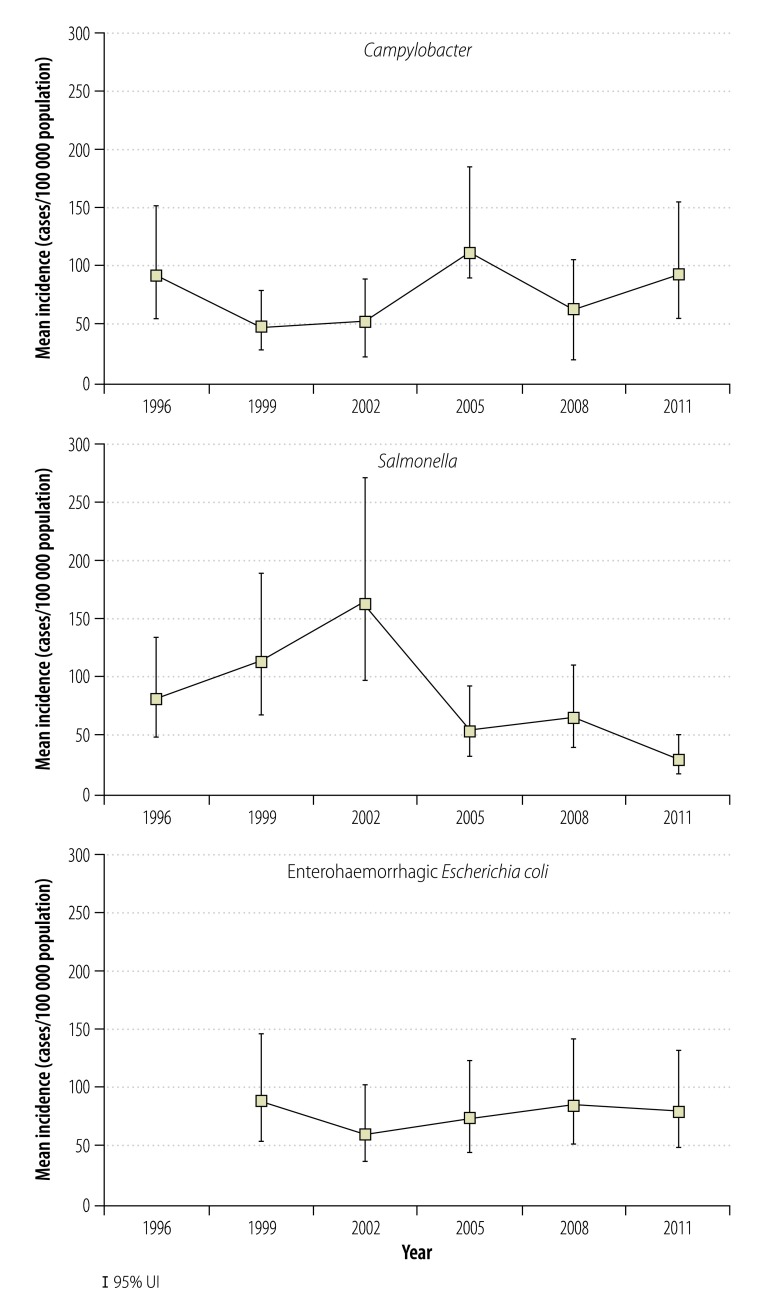
Estimates of the incidence of foodborne disease caused by *Campylobacter*, *Salmonella* or enterohaemorrhagic *Escherichia coli*, Japan, 2011

Table 1 shows the incidence of gastroenteritis caused by foodborne *Campylobacter*, *Salmonella* or EHEC reported in the routine surveillance data, and the corresponding – much higher – adjusted incidences that we estimated using the pyramid reconstruction method. Fig. 2 shows the estimated annual incidence of acute gastroenteritis caused by foodborne *Campylobacter*, *Salmonella* or EHEC between 1996 or 1999 and 2011. Over this period, there was no clear trend in the incidence of acute gastroenteritis caused by foodborne *Campylobacter* or EHEC but the incidence of gastroenteritis caused by foodborne *Salmonella* appeared to fall substantially after 2002.

**Table 1:** Estimated incidences of acute gastroenteritis, Japan, 2011

Data source	Causative agent	Estimated no. of cases	Estimated incidence, cases per 100 000 population (95% UI)
Food poisoning statistics	*Campylobacter* spp.	2341	1.8 (1.1–2.8)
	*Salmonella* sp.	3068	2.4 (1.5–3.6)
	EHEC	714	0.6 (0.2–1.3)
Pyramid reconstruction	*Campylobacter* spp.	118 502	92.5 (55.2–154.5)
	*Salmonella* sp.	40 571	31.7 (19.2–51.8)
	EHEC	103 338	80.7 (49.5–133.1)

EHEC: enterohaemorrhagic *Escherichia coli*; UI: uncertainty interval.

UI: uncertainty intervals.

### Disease burdens

Table 2 summarizes the experts’ estimates of the proportions of the acute gastroenteritis incidence that can be attributed to foodborne transmission and other pathways. Table 2 also shows the corresponding Bayesian factors used to adjust for seasonality, physician visits and stool examination – i.e. the denominators of Equation 1, Equation 2 and Equation 3.

**Table 2:** Estimated proportions of gastroenteritis cases resulting from foodborne transmission and other pathways, Japan, 2011

Causative agent	No. of experts^a^	Transmission pathway, % (95% UI)						Bayesian adjustment factor (95% UI)
		Food	Environment	Animal–human	Human–human		Travel	
*Campylobacter* spp.	15	82.0 (78.5–85.5)	8.3 (6.7–10.1)	3.1 (2.1–4.3)	0.2 (0.0–0.5)	6.4 (5.0–8.0)		0.17 (0.08–0.32)
*Salmonella* sp.	14	79.3 (74.7–84.0)	2.7 (1.7–3.8)	10.1 (8.4–12.0)	3.4 (2.4–4.7)	4.5 (3.2–5.9)		0.26 (0.12–0.47)
EHEC	20	77.6 (73.4–81.8)	4.0 (2.8–5.3)	8.5 (6.9–10.4)	6.0 (4.6–7.6)	3.9 (2.8–5.29		2.23 (0.97–4.00)

EHEC: enterohaemorrhagic *Escherichia coli*; UI: uncertainty interval.

^a^ These experts were asked to estimate the proportions of gastroenteritis cases resulting from each transmission pathway.

**Fig. 3:**
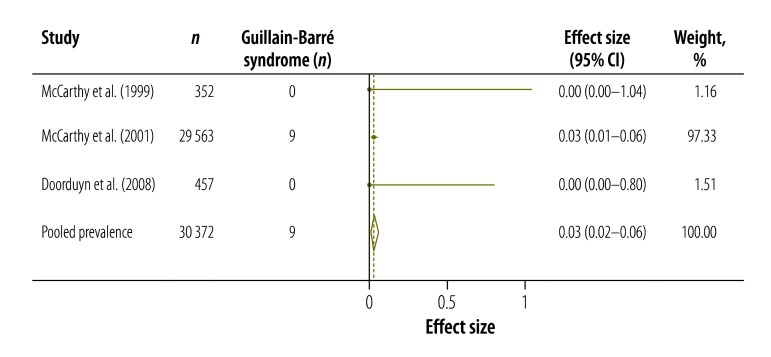
*Campylobacter* spp. associated cases of Guillain-Barré syndrome, 1999–2008

**Fig. 4:**
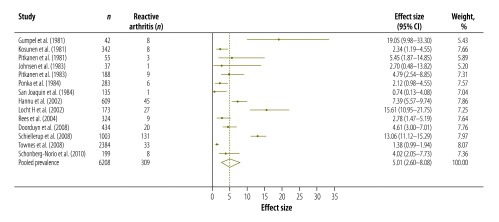
*Campylobacter* spp. associated cases of reactive arthritis, 1981–2010

**Fig. 5:**
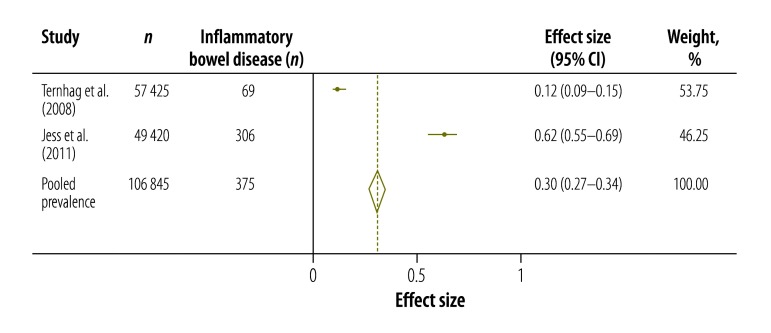
*Campylobacter* spp. associated cases of inflammatory bowel disease, 2008–2011

**Fig. 6:**
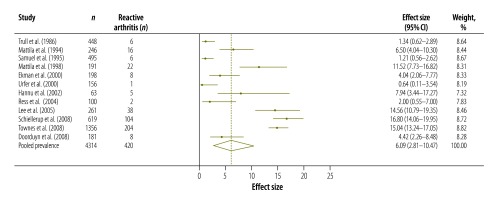
*Salmonella* sp. associated cases of reactive arthritis, 1986–2008

**Fig. 7:**
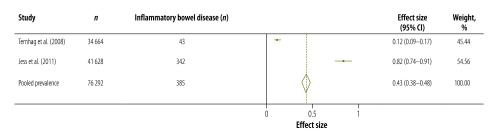
*Salmonella* sp. associated cases of inflammatory bowel disease, 2008–2011

**Fig. 8:**
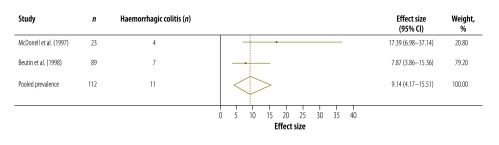
Enterohaemorrhagic *Escherichia coli*-associated cases of haemorrhagic colitis, 1997–1998

**Fig. 9:**
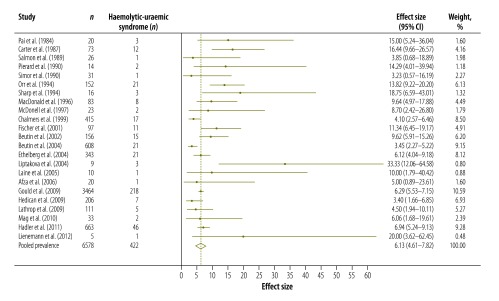
Enterohaemorrhagic *Escherichia coli*-associated cases of haemolytic-uraemic syndrome, 1984–2012

Table 3 shows the results of our systematic review and meta-analysis of the prevalence of various complications that may occur after infection with *Campylobacter*, *Salmonella* or EHEC (Fig. 3, Fig. 4, Fig. 5, Fig. 6, Fig. 7, Fig. 8 and Fig. 9; all available at: http://www.who.int/bulletin/volumes/93/08-/14-148056). The attributable proportions – i.e. the percentages of the cases of the sequelae that could be attributed to one of our pathogens of interest – varied from 0.03%, for Guillain-Barré syndrome and *Campylobacter*, to 9.14%, for haemorrhagic colitis and EHEC.

**Table 3:** Proportions of cases of sequelae attributable to *Campylobacter* spp., *Salmonella* sp. or enterohaemorrhagic *Escherichia coli*

Pathogen, sequelae	Attributable proportion, % of cases of sequelae, (95% UI)	No. of studies	Country
***Campylobacter* spp.**			
Guillain-Barré syndrome	0.03 (0.02–0.06)	3	Netherlands, Sweden
Inflammatory bowel disease	0.30 (0.27–0.34)	2	Denmark, Sweden
Reactive arthritis	5.01 (2.60–8.08)	14	Denmark, Finland, Netherlands, Norway, United Kingdom, USA
***Salmonella* sp.**			
Inflammatory bowel disease	0.43 (0.38–0.48)	2	Denmark, Sweden
Reactive arthritis	6.09 (2.81–10.47)	12	Australia, Denmark, Finland, Netherlands, Switzerland, United Kingdom, USA
**EHEC**			
Haemorrhagic colitis	9.14 (4.17–15.51)	2	Germany, United Kingdom
Haemolytic uraemic syndrome	6.13 (4.61–7.82)	23	Austria, Belgium, Canada, Denmark, Finland, Germany, Hungary, Slovakia, United Kingdom, USA

EHEC: enterohaemorrhagic *Escherichia coli*; UI: uncertainty interval; USA: United States of America.

Sources: Data drawn from the results of identified studies.^21^^–^^69^

CI: confidence interval.

Notes: Heterogeneity, *I^2^*: 0.0%. Logistic normal random-effects model was used to test effect size *z* = −24.37; *P* = 0.000.

CI: confidence interval.

Notes: Heterogeneity, *I^2^*: 94.7%. Freeman-Tukey double arcsine transformation was used to test effect size *z* = 6.13; *P* = 0.000.

CI: confidence interval.

Notes: Heterogeneity, *I^2^*: 0%. Freeman-Tukey double arcsine transformation was used to test effect size *z* = 34.65; *P* = 0.013.

CI: confidence interval.

Notes: Heterogeneity, *I^2^*: 96.0%. Freeman-Tukey double arcsine transformation was used to test effect size *z* = 5.43; *P* = 0.000.

CI: confidence interval.

Notes: Heterogeneity, *I^2^*: 0%. Freeman-Tukey double arcsine transformation was used to test effect size *z* = 34.9; *P* = 0.029.

CI: confidence interval.

Notes: Heterogeneity, *I^2^*: 43.8%. Freeman-Tukey double arcsine transformation was used to test effect size *z* = 3.60; *P* = 0.000.

CI: confidence interval.

Notes: Heterogeneity, *I^2^*: 63.4%. Freeman-Tukey double arcsine transformation was used to test effect size *z* = 12.19; *P* = 0.000.

Table 4 summarizes the numbers of deaths recorded in Japan in 2011 that were attributed to gastroenteritis caused by foodborne *Campylobacter*, *Salmonella* or EHEC or to the related complications. No deaths were attributed to gastroenteritis caused by *Campylobacter*, reactive arthritis or haemorrhagic colitis.

**Table 4:** Burdens posed by foodborne diseases caused by *Campylobacter* spp., *Salmonella* sp. or enterohaemorrhagic *Escherichia coli*, Japan, 2011

Causative agent, condition	Incidence, cases (95% UI)	Fatal cases	Years of illness	Disability weight	Burden metrics			YLD/DALY (%)
					YLD (95% UI)	YLL (95% UI)	DALY (95% UI)	
***Campylobacter* spp.**								
Gastroenteritis	118 502 (70 654–197 823)	–	–	–	122	0	122	
Visiting a general practitioner	4 833 (3 439–7 156)	0	0.03	0.39	50 (42–66)	0	50 (42–66)	100.0
Not visiting a general practitioner	114 219 (67 864–190 644)	0	0.01	0.07	72 (42–122)	0	72 (42–122)	100.0
Mild Guillain-Barré syndrome	30 (14–60)	0	1.00	0.25	7 (5–12)	0	7 (5–12)	100.0
Severe Guillain-Barré syndrome	5 (3–11)	1	29.26	0.16	29 (13–57)	12 (6–21)	42 (24–69)	69.0
Reactive arthritis	6 087 (2 956–11 156)	0	0.61	0.14	520 (257–952)	0	520 (257–952)	100.0
Inflammatory bowel disease	452 (93–1 051)	4	44.36	0.26	5 261(1 095–12 393)	83 (31–150)	5 344 (1 173–12 475)	98.4
Total	–	–	–	–	6003 (1 651–13 687)	96 (42–160)	6 099 (1 745–13 778)	98.4
***Salmonella* sp.**								
Gastroenteritis	40 571 (24 607–66 382)	–	–	–	70	122	192	
Visiting a general practitioner	3 866 (3 411–4 658)	3	0.03	0.39	47 (42–56)	122 (8–292)	169 (52–338)	27.8
Not visiting a general practitioner	36 667 (21 237–62 597)	0	0.02	0.07	23 (13–37)	0	23 (13–37)	100.0
Reactive arthritis	2 556 (1 190–4 774)	0	0.61	0.15	227 (119–390)	0	227 (119–390)	100.0
Inflammatory bowel disease	202 (36–481)	2	50.52	0.26	2652(492–6 211)	38 (13–69)	2 690 (522–6 236)	98.6
Total	–	–	–	–	2979 (753–6 795)	166 (49–350)	3 145 (906–6 950)	94.7
**EHEC**								
Gastroenteritis	103 338 (63 419–170 419)	–	–	–	75	130	205	
Visiting a general practitioner	2 064 (1 955–2 175)	10	0.02	0.39	12 (11–13)	130 (53–232)	142 (65–244)	8.5
Not visiting a general practitioner	101 982 (60 428–169 268)	0	0.01	0.07	63 (38–96)	0	63 (38–96)	100.0
Haemorrhagic colitis	229 (115–361)	0	0.02	0.39	1 (1–2)	0	1 (1–2)	100.0
Haemolytic uraemic syndrome^a^	132 (108–155)	3	NA	NA	133 (109–159)	108 (42–196)	240 (169–326)	55.4
Total	–	–	–	–	211 (171–266)	252 (129–395)	463 (325–606)	45.6

DALY: disability-adjusted life-years; EHEC: enterohaemorrhagic *Escherichia coli*; NA: not available; UI: uncertainty interval; YLD: years lived with disability; YLL: years of life lost.

^a^ Every case was estimated to correspond to 1.05 years lived with disability.^17^

Sources: years of illness and disability weights were based on values provided by Van Lier and Havelaar^17^ and Kemmeren et al.^18^

Table 4 also presents disability weights, disease durations, estimated incidences and disease burdens in terms of DALYs. Most of the overall disease burden posed by foodborne *Campylobacter*, *Salmonella* or EHEC was the result of a relatively small number of complications.

## Discussion

Our study provides national estimates of incidence, deaths and disease burden in DALYs, caused by *Campylobacter*, *Salmonella* and EHEC in Japan in 2011.

Estimates of annual incidence were approximately 92.5, 31.7 and 80.7 cases per 100 000 population for gastroenteritis caused by foodborne *Campylobacter*, *Salmonella* and EHEC, respectively. These estimates were many-fold higher than the values indicated by the results of routine surveillance, which ranged from 0.6 to 2.4 cases per 100 000 population. In 2011 at least, Japan’s routine surveillance system for foodborne diseases appeared to grossly underreport the incidence of acute gastroenteritis caused by our pathogens of interest. One probable cause of such underreporting is that the surveillance system focuses on clusters, outbreaks and other large public health events and usually ignores individual sporadic cases.^73^

Our estimate of the annual incidence of gastroenteritis caused by foodborne *Campylobacter* appears relatively low for a high-income country. Previous estimates of such incidence in a high-income country have ranged from 440 per 100 000, in the United States in 2006, to 930 per 100 000, in the United Kingdom in 2008–2009.^74^ Apparent geographical variation in the incidence of such disease may partly reflect between-country and between-study differences in the surveillance methods employed. In some countries, population-based cohort studies – e.g. the Sensor study in the Netherlands and two Infectious Intestinal Disease studies in the United Kingdom^75^^–^^77^ – are being used. In Australia, Canada and the United States, a surveillance pyramid method that included information on hospital visits and laboratory-confirmed cases is being employed.^78^^–^^82^ Harmonization of methods will be necessary if we are to make meaningful comparisons of incidence estimates between countries and over time. The results of this pilot study will hopefully help FERG to improve its recommendations and the production of comparable, consistent estimates of the incidences of foodborne diseases.

In our study, to address the potential bias resulting from the one-day hospital reporting period and the inclusion only of laboratory-confirmed cases, we applied a pyramid reconstruction technique similar to that used in previous research.^78^^–^^82^ Although this technique allows some adjustment for seasonality, care-seeking and diagnostic factors, it has several limitations. First, we estimated the age-specific incidence of gastroenteritis based on food poisoning statistics from a passive surveillance system, that tends to miss sporadically occurring cases.^83^ To make the estimation of the number of cases occurring annually in each age group more accurate, active surveillance – via national surveys or a population-based surveillance network – would be needed.

Second, we restricted the sequelae we investigated to those previously identified in a European study. In Japan, there may be different or more complications than observed in Europe.

Third, we based some of our estimation process on a systematic review of sequelae from other countries, where the epidemiology of foodborne disease may differ from that in Japan – e.g. because of the geographical variation in dietary habits.

Fourth, the validity and comparability of the disability weights that we used may be limited. In the field of foodborne disease, information on disability weights for specific complications is scarce. Hopefully, relevant data will soon be provided by FERG.^72^

Finally, our estimates of the proportion of gastroenteritis resulting from foodborne transmission were based on expert opinion instead of empirical data. The size of the so-called foodborne fraction appears to vary markedly depending on the country involved. Such a large variation may be due to differences in dietary habit, consumer tastes, food processing and food safety – but may also reflect differences in the methods used to investigate transmission pathways.

The approach recommended by FERG appears useful for understanding the magnitude of foodborne diseases, prioritizing food safety interventions and policies and harmonizing methods for the estimation of the foodborne disease burden.

## References

[1] Newell DG, Koopmans M, Verhoef L, Duizer E, Aidara-Kane A, Sprong H, et al. Food-borne diseases - the challenges of 20 years ago still persist while new ones continue to emerge. Int J Food Microbiol. 2010 5 30;139 Suppl 1:S3–15. 10.1016/j.ijfoodmicro.2010.01.021 20153070 PMC7132498

[2] Havelaar AH, Cawthorne A, Angulo F, Bellinger D, Corrigan T, Cravioto A, et al. WHO initiative to estimate the global burden of foodborne diseases. Lancet. 2013;381:59–59. 10.1016/S0140-6736(13)61313-6

[3] WHO initiative to estimate the global burden of foodborne diseases. First formal meeting of the Foodborne Disease Burden Epidemiology Reference Group (FERG). Geneva: World Health Organization; 2012. Available from: www.who.int/foodsafety/publications/foodborne_disease/FERG_Nov07.pdf [cited 2014 Sep 15].

[4] Polinder S, Haagsma JA, Stein C, Havelaar AH. Systematic review of general burden of disease studies using disability-adjusted life years. Popul Health Metr. 2012;10(1):21. 10.1186/1478-7954-10-21 23113929 PMC3554436

[5] Haagsma JA, Polinder S, Stein CE, Havelaar AH. Systematic review of foodborne burden of disease studies: quality assessment of data and methodology. Int J Food Microbiol. 2013 8 16;166(1):34–47. 10.1016/j.ijfoodmicro.2013.05.029 23827806

[6] Ono T, Shibuya K. Policy situation analysis of the Japanese food safety system. Tokyo: Ministry of Health, Labour and Welfare; 2012.

[7] Food poisoning statistics (data for foodborne disease outbreaks) [Internet]. Tokyo: Ministry of Health, Labour and Welfare; 2015. Available from: http://www.mhlw.go.jp/toukei/list/112-1.html [cited 2014 Aug 10]. Japanese.

[8] Infectious diseases weekly survey [Internet]. Tokyo: National Institute of Infectious Diseases; 1998. Available from: http://www.nih.go.jp/niid/en/ [cited 2014 Aug 10].

[9] Infectious agents surveillance report [Internet]. Tokyo: National Institute of Infectious Diseases; 1998. Available from: http://www.nih.go.jp/niid/en/iasr-e.html [cited 2014 Aug 10].

[10] The patient survey [Internet]. Tokyo: Ministry of Health, Labour and Welfare; 2015. Available from: http://www.mhlw.go.jp/toukei/list/10-20.html [cited 2014 Aug 10]. Japanese.

[11] Vital statistics (data for Japanese demographic situation) [Internet]. Tokyo: Ministry of Health, Labour and Welfare; 2011. Available from: http://www.e-stat.go.jp/SG1/estat/OtherList.do?bid=000001041646&cycode=7[cited 2014 Aug 10]. Japanese.

[12] Freeman J, Hutchison GB. Prevalence, incidence and duration. Am J Epidemiol. 1980 11;112(5):707–23. 6969024 10.1093/oxfordjournals.aje.a113043

[13] Kubota K, Iwasaki E, Inagaki S, Nokubo T, Sakurai Y, Komatsu M, et al. The human health burden of foodborne infections caused by *Campylobacter*, *Salmonella*, and *Vibrio parahaemolyticus* in Miyagi Prefecture, Japan. Foodborne Pathog Dis. 2008 10;5(5):641–8. 10.1089/fpd.2008.0092 18851675

[14] Kubota K, Kasuga F, Iwasaki E, Inagaki S, Sakurai Y, Komatsu M, et al. Estimating the burden of acute gastroenteritis and foodborne illness caused by *Campylobacter*, *Salmonella*, and *Vibrio parahaemolyticus* by using population-based telephone survey data, Miyagi Prefecture, Japan, 2005 to 2006. J Food Prot. 2011 10;74(10):1592–8. 10.4315/0362-028X.JFP-10-387 22004803

[15] Gelman A, Carlin JB, Stern HS, Dunson DB, Vehtari A, Rubin DB. Bayesian data analysis. 3rd ed Boca Rotan: Chapman and Hall; 2013.

[16] Havelaar AH, Galindo AV, Kurowicka D, Cooke RM. Attribution of foodborne pathogens using structured expert elicitation. Foodborne Pathog Dis. 2008 10;5(5):649–59. 10.1089/fpd.2008.0115 18687052

[17] Van Lier EA, Havelaar AH. Disease burden of infectious diseases in Europe: a pilot study (RIVM report 215001001). Bilthoven: National Institute for Public Health and the Environment; 2007.

[18] Kemmeren JM, Mangen MJJ. van Duynhoven YTHP, Havelaar AH. Priority setting of foodborne pathogens: disease burden and costs of selected enteric pathogens (RIVM report 330080001). Bilthoven: National Institute for Public Health and the Environment; 2006.

[19] Momose Y, Ota E, Shibuya K. Systematic review for foodborne diseases caused by *Salmonella* sp. and EHEC. Tokyo: Ministry of Health, Labour and Welfare; 2012.

[20] Wells GA, Shea B, O’Connell D, Peterson J, Welch V, Losos M, et al. The Newcastle-Ottawa Scale (NOS) for assessing the quality of nonrandomised studies in meta-analyses. Ottawa: Ottawa Hospital Research Institute; 2014.Available from: http://www.ohri.ca/programs/clinical_epidemiology/oxford.asp. [cited 2014 Feb 2014].

[21] McCarthy N, Andersson Y, Jormanainen V, Gustavsson O, Giesecke J. The risk of Guillain-Barré syndrome following infection with Campylobacter jejuni. Epidemiol Infect. 1999 2;122(1):15–7. 10.1017/S0950268898001861 10098780 PMC2809582

[22] McCarthy N, Giesecke J. Incidence of Guillain-Barré syndrome following infection with Campylobacter jejuni. Am J Epidemiol. 2001 3 15;153(6):610–4. 10.1093/aje/153.6.610 11257070

[23] Doorduyn Y, Van Pelt W, Siezen CL, Van Der Horst F, Van Duynhoven YT, Hoebee B, et al. Novel insight in the association between salmonellosis or campylobacteriosis and chronic illness, and the role of host genetics in susceptibility to these diseases. Epidemiol Infect. 2008 9;136(9):1225–34. 10.1017/S095026880700996X 18062835 PMC2870923

[24] Gumpel JM, Martin C, Sanderson PJ. Reactive arthritis associated with campylobacter enteritis. Ann Rheum Dis. 1981 2;40(1):64–5. 10.1136/ard.40.1.64 7469527 PMC1000658

[25] Kosunen TU, Pönkä A, Kauranen O, Martio J, Pitkänen T, Hortling L, et al. Arthritis associated with Campylobacter jejuni enteritis. Scand J Rheumatol. 1981;10(2):77–80. 10.3109/03009748109095276 7244582

[26] Pitkänen T, Pettersson T, Pönkä A, Kosunen TU. Clinical and serological studies in patients with Campylobacter fetus ssp. jejuni infection: I. Clinical findings. Infection. 1981;9(6):274–8. 10.1007/BF01640990 7333678

[27] Johnsen K, Ostensen M, Melbye AC, Melby K. HLA-B27-negative arthritis related to Campylobacter jejuni enteritis in three children and two adults. Acta Med Scand. 1983;214(2):165–8. 10.1111/j.0954-6820.1983.tb08589.x 6605028

[28] Pitkänen T, Pönkä A, Pettersson T, Kosunen TU. Campylobacter enteritis in 188 hospitalized patients. Arch Intern Med. 1983 2;143(2):215–9. 10.1001/archinte.1983.00350020033007 6824388

[29] Pönkä A, Pitkänen T, Sarna S, Kosunen TU. Infection due to Campylobacter jejuni: a report of 524 outpatients. Infection. 1984 May-Jun;12(3):175–8. 10.1007/BF01640893 6469363

[30] San Joaquin VH, Welch DF. Campylobacter enteritis. A 3-year experience. Clin Pediatr (Phila). 1984 6;23(6):311–6. 10.1177/000992288402300601 6609793

[31] Hannu T, Mattila L, Rautelin H, Pelkonen P, Lahdenne P, Siitonen A, et al. Campylobacter-triggered reactive arthritis: a population-based study. Rheumatology (Oxford). 2002 3;41(3):312–8. 10.1093/rheumatology/41.3.312 11934969

[32] Locht H, Krogfelt KA. Comparison of rheumatological and gastrointestinal symptoms after infection with Campylobacter jejuni/coli and enterotoxigenic Escherichia coli. Ann Rheum Dis. 2002 5;61(5):448–52. 10.1136/ard.61.5.448 11959770 PMC1754099

[33] Rees JR, Pannier MA, McNees A, Shallow S, Angulo FJ, Vugia DJ. Persistent diarrhea, arthritis, and other complications of enteric infections: a pilot survey based on California FoodNet surveillance, 1998–1999. Clin Infect Dis. 2004 4 15;38(s3) Suppl 3:S311–7. 10.1086/381601 15095204

[34] Schiellerup P, Krogfelt KA, Locht H. A comparison of self-reported joint symptoms following infection with different enteric pathogens: effect of HLA-B27. J Rheumatol. 2008 3;35(3):480–7. 18203313

[35] Townes JM, Deodhar AA, Laine ES, Smith K, Krug HE, Barkhuizen A, et al. Reactive arthritis following culture-confirmed infections with bacterial enteric pathogens in Minnesota and Oregon: a population-based study. Ann Rheum Dis. 2008 12;67(12):1689–96. 18272671 10.1136/ard.2007.083451

[36] Schönberg-Norio D, Mattila L, Lauhio A, Katila ML, Kaukoranta SS, Koskela M, et al. Patient-reported complications associated with Campylobacter jejuni infection. Epidemiol Infect. 2010 7;138(7):1004–11. 10.1017/S0950268809991099 19887016

[37] Ternhag A, Törner A, Svensson A, Ekdahl K, Giesecke J. Short- and long-term effects of bacterial gastrointestinal infections. Emerg Infect Dis. 2008 1;14(1):143–8. 10.3201/eid1401.070524 18258094 PMC2600169

[38] Jess T, Simonsen J, Nielsen NM, Jørgensen KT, Bager P, Ethelberg S, et al. Enteric Salmonella or Campylobacter infections and the risk of inflammatory bowel disease. Gut. 2011 3;60(3):318–24. 10.1136/gut.2010.223396 21193449

[39] Trull AK, Eastmond CJ, Panayi GS, Reid TM. Salmonella reactive arthritis: serum and secretory antibodies in eight patients identified after a large outbreak. Br J Rheumatol. 1986 2;25(1):13–9. 10.1093/rheumatology/25.1.13 3942838

[40] Mattila L, Leirisalo-Repo M, Koskimies S, Granfors K, Siitonen A. Reactive arthritis following an outbreak of Salmonella infection in Finland. Br J Rheumatol. 1994 12;33(12):1136–41. 10.1093/rheumatology/33.12.1136 8000742

[41] Samuel MP, Zwillich SH, Thomson GT, Alfa M, Orr KB, Brittain DC, et al. Fast food arthritis–a clinico-pathologic study of post-Salmonella reactive arthritis. J Rheumatol. 1995 10;22(10):1947–52. 8991996

[42] Mattila L, Leirisalo-Repo M, Pelkonen P, Koskimies S, Granfors K, Siitonen A. Reactive arthritis following an outbreak of Salmonella Bovismorbificans infection. J Infect. 1998 5;36(3):289–95. 10.1016/S0163-4453(98)94243-8 9661939

[43] Ekman P, Kirveskari J, Granfors K. Modification of disease outcome in Salmonella-infected patients by HLA-B27. Arthritis Rheum. 2000 7;43(7):1527–34. 10.1002/1529-0131(200007)43:7<1527::AID-ANR17>3.0.CO;2-G 10902756

[44] Urfer E, Rossier P, Méan F, Krending MJ, Burnens A, Bille J, et al. Outbreak of Salmonella braenderup gastroenteritis due to contaminated meat pies: clinical and molecular epidemiology. Clin Microbiol Infect. 2000 10;6(10):536–42. 10.1046/j.1469-0691.2000.00148.x 11168047

[45] Lee AT, Hall RG, Pile KD. Reactive joint symptoms following an outbreak of Salmonella typhimurium phage type 135a. J Rheumatol. 2005 3;32(3):524–7. 15742447

[46] McDonnell RJ, Rampling A, Crook S, Cockcroft PM, Wilshaw GA, Cheasty T, et al. An outbreak of Vero cytotoxin producing Escherichia coli O157 infection associated with takeaway sandwiches. Commun Dis Rep CDR Rev. 1997 12 12;7(13):R201–5. 9447785

[47] Beutin L, Zimmermann S, Gleier K. Human infections with Shiga toxin-producing Escherichia coli other than serogroup O157 in Germany. Emerg Infect Dis. 1998 Oct-Dec;4(4):635–9. 10.3201/eid0404.980415 9866741 PMC2640265

[48] Pai CH, Gordon R, Sims HV, Bryan LE. Sporadic cases of hemorrhagic colitis associated with Escherichia coli O157:H7. Clinical, epidemiologic, and bacteriologic features. Ann Intern Med. 1984 12;101(6):738–42. 10.7326/0003-4819-101-6-738 6388450

[49] Carter AO, Borczyk AA, Carlson JAK, Harvey B, Hockin JC, Karmali MA, et al. A severe outbreak of Escherichia coli O157:H7–associated hemorrhagic colitis in a nursing home. N Engl J Med. 1987 12 10;317(24):1496–500. 10.1056/NEJM198712103172403 3317047

[50] Salmon RL, Farrell ID, Hutchison JGP, Coleman DJ, Gross RJ, Fry NK, et al. A christening party outbreak of haemorrhagic colitis and haemolytic uraemic syndrome associated with Escherichia coli O 157.H7. Epidemiol Infect. 1989 10;103(2):249–54. 10.1017/S0950268800030600 2680545 PMC2249521

[51] Piérard D, Van Etterijck R, Breynaert J, Moriau L, Lauwers S. Results of screening for verocytotoxin-producing Escherichia coli in faeces in Belgium. Eur J Clin Microbiol Infect Dis. 1990 3;9(3):198–201. 10.1007/BF01963837 2186912

[52] Simor AE, Watt C, Low DE. The isolation rate of Escherichia coli 0157:H7 in Toronto and surrounding communities. Can J Infect Dis. 1990 Spring;1(1):23–7. 22553432 10.1155/1990/583209 PMC3327950

[53] Orr P, Lorencz B, Brown R, Kielly R, Tan B, Holton D, et al. An outbreak of diarrhea due to verotoxin-producing Escherichia coli in the Canadian Northwest Territories. Scand J Infect Dis. 1994;26(6):675–84. 10.3109/00365549409008635 7747090

[54] Sharp JC, Ritchie LD, Curnow J, Reid TM. High incidence of haemorrhagic colitis due to Escherichia coli O157 in one Scottish town: clinical and epidemiological features. J Infect. 1994 11;29(3):343–50. 10.1016/S0163-4453(94)91381-1 7884230

[55] MacDonald IA, Gould IM, Curnow J. Epidemiology of infection due to Escherichia coli O157: a 3-year prospective study. Epidemiol Infect. 1996 6;116(3):279–84. 10.1017/S0950268800052584 8666071 PMC2271426

[56] Chalmers RM, Parry SM, Salmon RL, Smith RMM, Willshaw GA, Cheasty T. The surveillance of vero cytotoxin-producing Escherichia coli O157 in Wales, 1990 to 1998. Emerg Infect Dis. 1999 Jul-Aug;5(4):566–9. 10.3201/eid0504.990422 10458968 PMC2627734

[57] Fischer H, König P, Dierich MP, Allerberger F. Hemolytic-uremic syndrome surveillance to monitor trends in infection with Escherichia coli O157 and non-O157 enterohemorrhagic E. coli in Austria. Pediatr Infect Dis J. 2001 3;20(3):316–8. 10.1097/00006454-200103000-00021 11303839

[58] Beutin L, Kaulfuss S, Cheasty T, Brandenburg B, Zimmermann S, Gleier K, et al. Characteristics and association with disease of two major subclones of Shiga toxin (Verocytotoxin)-producing strains of Escherichia coli (STEC) O157 that are present among isolates from patients in Germany. Diagn Microbiol Infect Dis. 2002 12;44(4):337–46. 10.1016/S0732-8893(02)00474-1 12543538

[59] Beutin L, Krause G, Zimmermann S, Kaulfuss S, Gleier K. Characterization of Shiga toxin-producing Escherichia coli strains isolated from human patients in Germany over a 3-year period. J Clin Microbiol. 2004 3;42(3):1099–108. 10.1128/JCM.42.3.1099-1108.2004 15004060 PMC356890

[60] Ethelberg S, Olsen KEP, Scheutz F, Jensen C, Schiellerup P, Enberg J, et al. Virulence factors for hemolytic uremic syndrome, Denmark. Emerg Infect Dis. 2004 5;10(5):842–7. 10.3201/eid1005.030576 15200817 PMC3323205

[61] Liptakova A, Siegfried L, Rosocha J, Podracka L, Bogyiova E, Kotulova D. A family outbreak of haemolytic uraemic syndrome and haemorrhagic colitis caused by verocytotoxigenic Escherichia coli O157 from unpasteurised cow’s milk in Slovakia. Clin Microbiol Infect. 2004 6;10(6):576–8. 10.1111/j.1469-0691.2004.00900.x 15191389

[62] Laine ES, Scheftel JM, Boxrud DJ, Vought KJ, Danila RN, Elfering KM, et al. Outbreak of Escherichia coli O157:H7 infections associated with nonintact blade-tenderized frozen steaks sold by door-to-door vendors. J Food Prot. 2005 6;68(6):1198–202. 15954707 10.4315/0362-028x-68.6.1198

[63] Afza M, Hawker J, Thurston H, Gunn K, Orendi J. An outbreak of Escherichia coli O157 gastroenteritis in a care home for the elderly. Epidemiol Infect. 2006 12;134(6):1276–81. 10.1017/S0950268806006546 16740198 PMC2870527

[64] Gould LH, Demma L, Jones TF, Hurd S, Vugia DJ, Smith K, et al. Hemolytic uremic syndrome and death in persons with Escherichia coli O157:H7 infection, foodborne diseases active surveillance network sites, 2000–2006. Clin Infect Dis. 2009 11 15;49(10):1480–5. 10.1086/644621 19827953

[65] Hedican EB, Medus C, Besser JM, Juni BA, Koziol B, Taylor C, et al. Characteristics of O157 versus non-O157 Shiga toxin-producing Escherichia coli infections in Minnesota, 2000–2006. Clin Infect Dis. 2009 8 1;49(3):358–64. 10.1086/600302 19548834

[66] Lathrop S, Edge K, Bareta J. Shiga toxin-producing Escherichia coli, New Mexico, USA, 2004–2007. Emerg Infect Dis. 2009 8;15(8):1289–91. 10.3201/eid1508.081616 19751594 PMC2815966

[67] Mag T, Nógrády N, Herpay M, Tóth I, Rozgonyi F. Characterisation of verotoxin-producing Escherichia coli strains isolated from human patients in Hungary over a 7-year period. Eur J Clin Microbiol Infect Dis. 2010 2;29(2):249–52. 10.1007/s10096-009-0836-z 19957004

[68] Hadler JL, Clogher P, Hurd S, Phan Q, Mandour M, Bemis K, et al. Ten-year trends and risk factors for non-O157 Shiga toxin-producing Escherichia coli found through Shiga toxin testing, Connecticut, 2000–2009. Clin Infect Dis. 2011 8 1;53(3):269–76. 10.1093/cid/cir377 21765075

[69] Lienemann T, Salo E, Rimhanen-Finne R, Rönnholm K, Taimisto M, Hirvonen JJ, et al. Shiga toxin-producing Escherichia coli serotype O78:H(-) in family, Finland, 2009. Emerg Infect Dis. 2012 4;18(4):577–81. 10.3201/eid1804.111310 22469631 PMC3309701

[70] Barendregt JJ, Doi SA, Lee YY, Norman RE, Vos T. Meta-analysis of prevalence. J Epidemiol Community Health. 2013 11 1;67(11):974–8. 10.1136/jech-2013-203104 23963506

[71] Murray CJL, Acharya AK. Understanding DALYs (disability-adjusted life years). J Health Econ. 1997 12;16(6):703–30. 10.1016/S0167-6296(97)00004-0 10176780

[72] Murray CJL, Vos T, Lozano R, Naghavi M, Flaxman AD, Michaud C, et al. Disability-adjusted life years (DALYs) for 291 diseases and injuries in 21 regions, 1990–2010: a systematic analysis for the Global Burden of Disease Study 2010. Lancet. 2012 12 15;380(9859):2197–223. 10.1016/S0140-6736(12)61689-4 23245608

[73] Kumagai Y, Noda M, Kasuga F. New approaches for tackling foodborne infections. J Disaster Res. 2011;6(4):451–8.

[74] The global view of campylobacterosis: report and expert consultation. Geneva: World Health Organization; 2012. Available from: http://apps.who.int/iris/bitstream/10665/80751/1/9789241564601_eng.pdf[cited 2014 Aug 10].

[75] Wheeler JG, Sethi D, Cowden JM, Wall PG, Rodrigues LC, Tompkins DS, et al.; The Infectious Intestinal Disease Study Executive. Study of infectious intestinal disease in England: rates in the community, presenting to general practice, and reported to national surveillance. BMJ. 1999 4 17;318(7190):1046–50. 10.1136/bmj.318.7190.1046 10205103 PMC27838

[76] Tam CC, Rodrigues LC, Viviani L, Dodds JP, Evans MR, Hunter PR, et al.; IID2 Study Executive Committee. Longitudinal study of infectious intestinal disease in the UK (IID2 study): incidence in the community and presenting to general practice. Gut. 2012 1;61(1):69–77. 10.1136/gut.2011.238386 21708822 PMC3230829

[77] de Wit MA, Koopmans MP, Kortbeek LM, Wannet WJ, Vinjé J, van Leusden F, et al. Sensor, a population-based cohort study on gastroenteritis in the Netherlands: incidence and etiology. Am J Epidemiol. 2001 10 1;154(7):666–74. 10.1093/aje/154.7.666 11581101

[78] Angulo FJ, Voetsch AC, Vugia D, Hadler JL, Farley M, Hedberg C, et al. Determining the burden of human illness from food borne diseases. CDC’s emerging infectious disease program Food Borne Diseases Active Surveillance Network (FoodNet). Vet Clin North Am Food Anim Pract. 1998 3;14(1):165–72. 9532675 10.1016/s0749-0720(15)30287-5

[79] Scallan E, Hoekstra RM, Angulo FJ, Tauxe RV, Widdowson MA, Roy SL, et al. Foodborne illness acquired in the United States–major pathogens. Emerg Infect Dis. 2011 1;17(1):7–15. 10.3201/eid1701.P11101 21192848 PMC3375761

[80] Thomas MK, Majowicz SE, Sockett PN, Fazil A, Pollari F, Doré K, et al. Estimated numbers of community cases of illness due to *Salmonella*, *Campylobacter* and verotoxigenic *Escherichia coli*: pathogen-specific community rates. Can J Infect Dis Med Microbiol. 2006 7;17(4):229–34. 18382633 10.1155/2006/806874 PMC2095082

[81] Majowicz SE, Edge VL, Fazil A, McNab WB, Doré KA, Sockett PN, et al. Estimating the under-reporting rate for infectious gastrointestinal illness in Ontario. Can J Public Health. 2005 May-Jun;96(3):178–81. 15913079 10.1007/BF03403685 PMC6975884

[82] Hall G, Yohannes K, Raupach J, Becker N, Kirk M. Estimating community incidence of *Salmonella*, *Campylobacter*, and Shiga toxin-producing *Escherichia coli* infections, Australia. Emerg Infect Dis. 2008 10;14(10):1601–9. 10.3201/eid1410.071042 18826825 PMC2609882

[83] Okabe N, Sunagawa T. Study for improving the investigation of foodborne diseases. Tokyo: Ministry of Health, Labour and Welfare; 2013.

