# Corrigendum

**DOI:** 10.2471/BLT.15.101015

**Published:** 2015-10-01

In Volume 93, Issue 8, August 2015, page 541, equation (3) should read:


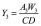


